# A new species of *Pionothele* from Gobabeb, Namibia (Araneae, Mygalomorphae, Nemesiidae)

**DOI:** 10.3897/zookeys.851.31802

**Published:** 2019-06-03

**Authors:** Jason E. Bond, Trip Lamb

**Affiliations:** 1 Department of Entomology & Nematology, University of California Davis, Davis, California, USA University of California Davis United States of America; 2 Department of Biology, East Carolina University, Greenville, North Carolina, USA East Carolina University Greenville United States of America

**Keywords:** Biodiversity, New species, Spider taxonomy, *
Pionothele
*, Nemesiidae, Mygalomorphae

## Abstract

The mygalomorph spider genus *Pionothele* Purcell, 1902 comprises two nominal species known only from South Africa. We describe here a new species, *Pionothelegobabeb***sp. n.**, from Namibia. This new species is currently only known from a very restricted area in the Namib Desert of western Namibia.

## Introduction

The nemesiid genus *Pionothele* Purcell, 1902 is a poorly known taxon comprising only two species described from southwestern South Africa. In Zonstein’s (2016) review of the genus, he redescribed and illustrated *P.straminea* Purcell, 1902 and described a second, new species *P.capensis* Zonstein, 2016. Similarities between female specimens of *Pionothele* and those in the genus *Spiroctenus*Simon 1889a suggest that some species described as the latter may be misidentified as the former (Zonstein 2016); consequently, *Pionothele* may be more widespread and diverse than is currently known. We describe herein a new species, *Pionothelegobabeb* sp. n., from the Namib Desert in central western Namibia; the type locality at the Gobabeb Research & Training Center is about120 km southeast of the Atlantic coastal city of Walvis Bay. The description of this new species extends the distribution of *Pionothele* significantly northward, indicating that the genus may contain considerable undescribed diversity, particularly in the intervening areas.

**Figure 1.**
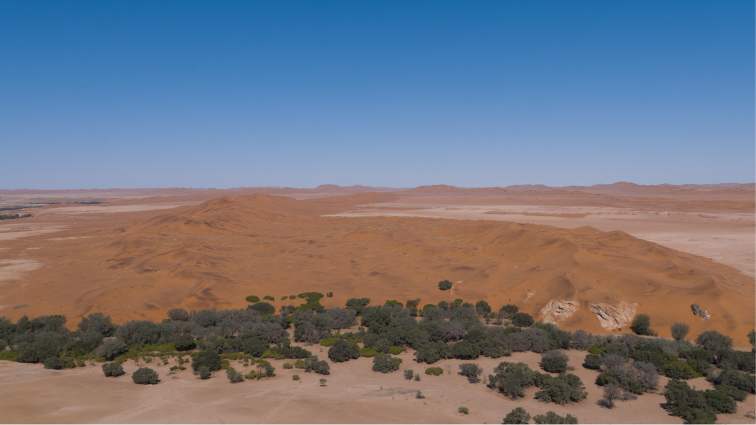
Aerial photograph of type locality. Kuiseb River bed in foreground (tree line); the interdune pitfall trap transect lies beyond the dunes (middle right of image).

**Habitat and ecology.** Fifteen males were collected in pitfall traps after a rain event at Gobabeb; specimens were observed along interdune and gravel plain transects – two of six habitats monitored by long-term pitfall trapping (Henschel et al. 2003). Gobabeb lies adjacent to the Kuiseb River, an ephemeral drainage where the northern terminus of the Namib Sand Sea abuts the gravel plains of the Central Namib. Here dune, riparian, and gravel plain habitats occur in close proximity. Figure 1 illustrates the Gobabeb collecting locality. The single female specimen was collected from a subterranean burrow on a sandy slope. All nominal species of *Pionothele* have been collected from dune ecosystems (or close proximity thereof).

**Species concept applied.** This new species of *Pionothele* is delineated using a traditional morphological species concept wherein species are defined as those populations with qualitative phenotypic characteristics that differ in a discrete manner from other populations or groups.

## Abbreviations, materials and methods

Institutional and quantitative morphological abbreviations used in this paper are defined as follows:

### Institutional

**BME** Bohart Museum of Entomology, Davis, California.

**NMN** National Museum of Namibia, Windhoek, Namibia

**CAS**California Academy of Sciences, San Francisco, California.

### Quantitative morphological features

The following features are explicitly defined and illustrated in Bond (2012):

**ANTd** number of teeth on the anterior margin of cheliceral fang furrow.

**Cl**, **Cw** carapace length and width. Carapace length taken along the midline dorsal-most posterior position to the anterofrontal edge of the carapace (chelicerae are not included in length). Carapace width taken at the widest point.

**AME**, **ALE**, **PME**, **PLE** anterior median, anterior lateral, posterior median, and posterior lateral eyes, respectively.

**LBl**, **LBw** labium length and width taken from the longest and widest points, respectively.

**PTl**, **PTw** male palpal tibia length and width.

**Bl** palpal bulb length from embolus tip to the bulb base, taken in the ventral plane at its longest point.

**PTLs**, **TBs** number of female prolateral patella and tibial spines leg III.

**STRl**, **STRw** sternum length and width. Sternum length from the base of the labium to its most posterior point. Width taken across the widest point, usually between legs II and III.

**PLS** posterior lateral spinneret

**TSrd**, **TSp**, **TSr** number of tibiaI spines on the distal most retrolateral, prolateral, and midline retrolateral positions.

**ITC** inferior tarsal claw

### Measurement, characterization, and illustration of morphological features

Format, descriptors, and morphological features measured/examined follows closely Bond (2012). Unique voucher numbers were assigned to all specimens (alphanumeric designations beginning with NMB); these data were added to each vial and can be used to cross-reference all images, measurements, and locality data. All measurements are given in millimeters and were made with a Leica MC205 dissecting microscope equipped with the Leica Analysis Suite Software. Lengths of leg articles were taken from the mid-proximal point of the articulation to the mid-distal point of the article (*sensu*Bond 2012, figs 11–16). Leg I and Leg IV article measurements are listed in the species description in the following order: femur, patella, tibia, metatarsus, tarsus. Carapace and leg coloration are described semi-quantitatively using Munsell® Color Charts (Windsor, NY) and are given using the color name and color notation (hue value/chroma).

Digital images of specimens were made using a BKPlus Digital Imaging System (Dun Inc.^TM^, Richmond, VA) where images were recorded at multiple focal planes and then assembled into a single focused image using Helicon Focus (Helicon Soft, Ltd., Ukraine). The female genital region was removed from the abdominal wall and tissues dissolved using trypsin; spermathecae were examined and photographed in the manner described above. Following Bond (2012), habitus illustrations were constructed from whole body images that were bisected, copied, and reflected in Adobe Photoshop (Adobe Systems, Inc.) to produce a roughly symmetrical image; the actual raw images are available upon request from the first author. Unless otherwise stated, scale bars = 1.0 mm.

### Locality data and georeferencing

Latitude and longitude for all collecting localities were recorded in the field using a Garmin Global Positioning System receiver (Garmin International Ltd., Olathe, KS) using WGS84 map datum.

## Taxonomy

### Family Nemesiidae Simon, 1889b

http://zoobank.org/638FB63E-DB51-4FB5-85AF-C04E81D3DBD7

urn:lsid:nmbe.ch:spiderfam:0007

#### Genus *Pionothele* Purcell, 1902

http://zoobank.org/4B5E1D34-582C-4259-BAE5-D5FE6AF68BEE

urn:lsid:nmbe.ch:spidergen:00127

*Pionothele* Purcell, 1902: 380 (type species by monotypy *Pionothelestraminea* male holotype from South Africa). – Tucker 1917: 117. – Raven 1985: 93.

##### Pionothele gobabeb sp. n.

Taxon classificationAnimaliaAraneaeNemesiidae

http://zoobank.org/87176CD8-22EB-4428-A293-80D16646EFD2

http://species-id.net/wiki/Pionothele_gobabeb

Figs 1 , 2–4 , 5–9

###### Type material.

Male holotype (NMB012_001; deposited in the BME) and additional male paratypes (one each deposited in the NMN, and CAS) from the Erongo Region, Namibia, in vicinity of Gobabeb Research & Training Center, along D1983 and Kuiseb River, – 23.56984 15.03984, coll. by J. Bond and T. Lamb 27.ix.2013.

###### Etymology.

The specific epithet is a noun taken in apposition and is in reference to the type locality.

###### Diagnosis.

**Figures 2–4.**
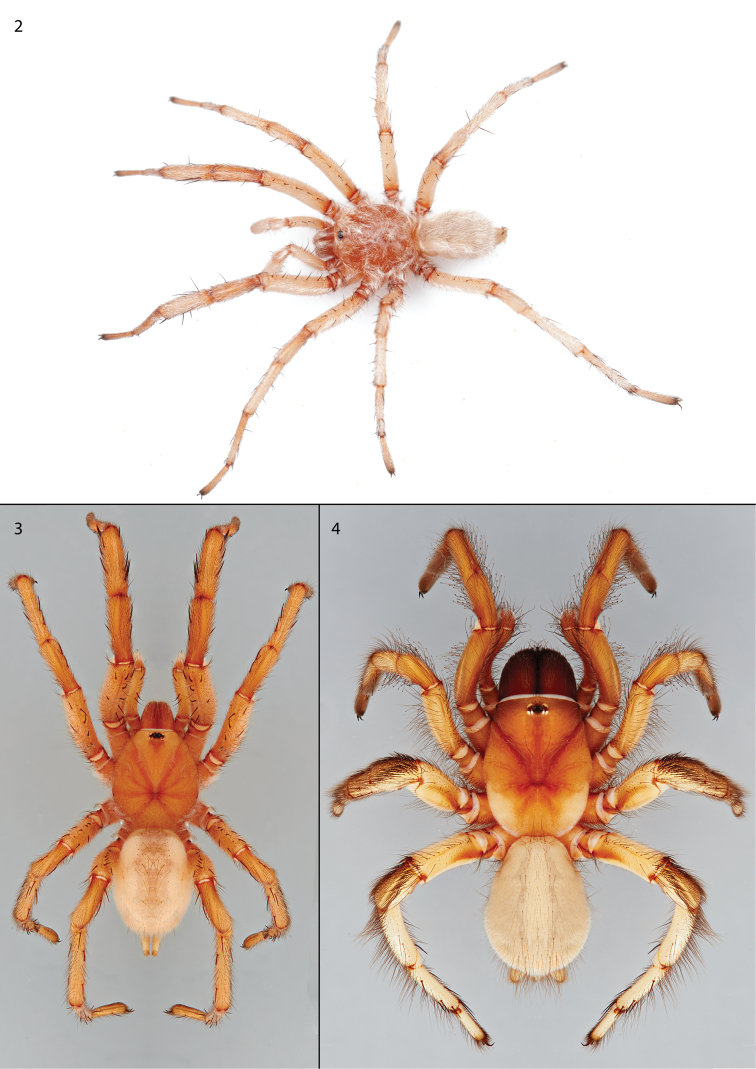
Habitus photograph and illustrations of *Pionothelegobabeb* sp. n. **2** Live male specimen **3** habitus digital illustration of male holotype specimen **4** habitus digital illustration of female.

**Figures 5–9.**
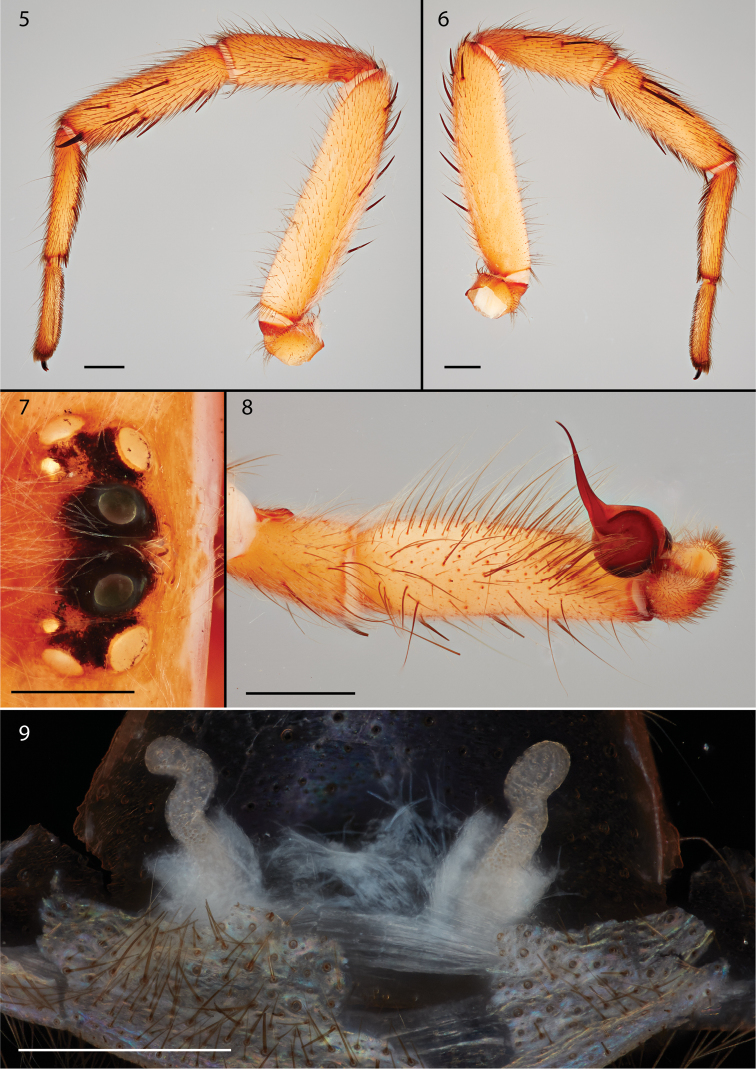
Photographic illustrations of male (holotype) and female *Pionothelegobabeb* sp. n. **5** male leg I and mating clasper, retrolateral view **6** male leg I and mating clasper, prolateral view **7** male eye group **8** male pedipalp distal segments and bulb **9** cleared spermathecae. Scale bar: 0.5 mm (**7, 8, 9**).

Male and female specimens (Figs 2–4) can be differentiated from the other two described species of *Pionothele* by having posterior median eyes that are reduced in size (Fig. 7), nearly half the diameter of the posterior lateral eyes and much smaller than the anterior median eyes. Like *P.capensis* the male palpal tibia is more slender than in *P.straminea* but like the latter lacks spines (Fig. 8); leg I has more mid-retrolateral spines than *P.capensis*, with a single large mid-distal spine and only two proximal prolateral spines (Figs 5, 6). Males and females both are very light in coloration similar to that of *P.straminea* (Figs 2–4), noted by Raven (1985) as “faded,” whereas the abdomen of *P.capensis* is pigmented and mottled. Spermathecal bulbs of *P.gobabeb* are moderately thin and sinuous whereas those illustrated for *P.capensis* are described as “wide and flattened” (Fig. 9); females also appear to have far fewer endite cuspules (25 vs 80).

###### Description of male holotype.

*Specimen preparation and condition.* Specimen preserved in 70% EtOH. Pedipalp, leg I removed, stored in vial with specimen. *General coloration in alcohol.* Carapace yellowish-red 5YR 4/6. Abdomen very pale brown 10YR 7/3. *Cephalothorax.* Carapace 7.58 long, 6.80 wide, very hirsute with fine white setae, pars cephalica slightly elevated. Fringe lacks heavy setae at posterior corners. Foveal groove deep, procurved.Tubercle absent. AER, PER slightly procurved. PME much smaller in diameter than AME, half the size of PLE. Sternum moderately setose, STRl 4.41, STRw 3.40. Posterior sternal sigilla small, round not contiguous; anterior sigilla pair smaller, placed at margin. ANTd comprising 5 large teeth; posterior margin with single row of 6 smaller teeth. Palpal endites, ~21 cuspules restricted to the anteroproximal margin, labium lacking cuspules, LBw 0.92, LBl 0.67. Rastellum absent. *Abdomen.* Moderately setose; apical segment of PLS short, triangular in shape. *Legs.* Leg I: 8.92, 4.62, 5.81, 4.16, 3.14; leg IV: 8.924, 3.31, 7.38, 6.95, 3.93. Light scopulae on all tarsi. Tarsus I with thin band of ~20 trichobothria. ITC legs I–III absent, leg IV small, sharply curved. Paired claws biserially dentate. Leg I spination pattern (Figs 5, 6); TSp 4, TSr 4, TSrd 1. *Pedipalp.*PTw 0.1.18, PTl 3.77, Bl 1.86. Embolus arises sharply from bulb, long thin tapered (Fig. 8).

###### Variation (n = 5).

**Cl** 6.18–7.59, 6.96±0.27; **Cw** 5.72–6.8, 6.24±0.21; **STRl** 3.56–4.41, 3.99±0.16; **STRw** 2.78–3.4, 3.1±0.12; **LBw** 0.88–1.11, 1.01±0.05; **LBl** 0.54–0.67, 0.62±0.02; **leg I**: 7.66–8.92, 8.45±0.24; 4.07–4.65, 4.39±0.13; 5.11–5.81, 5.39±0.13; 3.61–4.33, 4.03±0.12; 2.9–3.4, 3.14±0.09; **leg IV**: 7.96–8.92, 8.51±0.21; 2.75–3.72, 3.27±0.16; 5.98–7.38, 6.54±0.26; 5.5–6.98, 6.39±0.27; 3.36–3.93, 3.78±0.11; **PTl** 3.45–3.88, 3.72±0.08; **PTw** 0.88–1.18, 1.01±0.06; **Bl** 1.86–2.19, 2.02±0.07; **TSp** 2–4, 3.4±0.4; **TSr** 2–4, 3±0.32; **TSrd** 1–1, 1±0.

###### Description of non-type female (NMB012_001).

*Specimen preparation and condition.* Specimen preserved in same manner as male holotype. *Color.* Carapace yellowish red 5YR 4/6. Abdomen light yellowish-brown 10YR 6/4. *Cephalothorax.* Carapace 8.13 long, 6.08 wide, hirsute with fine white setae as in male; lacks fringe. Foveal groove deep and slightly recurved. Tubercle absent. AER very slightly procurved, PER straight to slightly recurved. AME reduced in size, smaller than PME. Sternum moderately setose, STRl 4.49, STRw 3.43. Posterior sigilla small, widely separated; medial anterior sigilla relatively small, positioned laterally. ANTd with 6 teeth with posterior margin comprising 4 teeth. Palpal endites, ~25 cuspules, restricted to the anterior margin endites; labium lacks cuspules, LBw 1.28, LBl 0.97. Rastellum absent. *Legs.* Leg I: 5.69, 3.19, 3.84, 3.09, 2.30; leg IV: 4.29, 3.58, 5.15, 4.46, 2.53. Dense scopulae tarsus/metatarsus of Legs I/II, tarsus/tibia of pedipalp. Tarsus I with ~18 trichobothria arranged in a relatively tight row. PTLs 4, TBs 2. ITC small, sharply precurved; paired claws biserially dentate. Preening combs absent. Female specimen has numerous setae on carapace and legs modified as spatulate (Fig. 4). Spermathecae bulbs thin and sinuous (Fig. 9). Apical segment of PLS short, domed.

###### Remarks.

The female specimen described herein is from a locality some distance from where the male specimens and male holotype/paratypes were collected (formally designated as the type locality). As such we do not describe the female as a paratype so as not to confuse the type locality or the identity of the species if the female specimen is eventually discovered to be a different species – acknowledging that mygalomorph spiders are known to be highly endemic with considerable species crypsis (see Bond & Stockman 2008). Nevertheless, we are reasonably confident that these specimens are conspecifics given similarities in morphology (e.g., size of the PMEs), habitat, and an explicit morphological species concept (applied herein).

###### Additional material examined.

Male specimens (12) collected in pitfall trips in vicinity of the type locality at Gobabeb, deposited in the BME. Single female specimen (NMB012_001) from the Erongo Region, Namibia, in vicinity of Intersection C39 and Huab River, – 20.36035 14.19186898, coll. J. Bond 19.ix.2013, deposited in BME.

###### Distribution.

Known only from the Erongo Region, Namibia.

## Supplementary Material

XML Treatment for Pionothele gobabeb
